# Low glycemic index therapy in children with sub-acute sclerosing panencephalitis (SSPE): an experience from a measles-endemic country

**DOI:** 10.3389/fnut.2023.1203144

**Published:** 2023-07-24

**Authors:** Shahnaz H. Ibrahim, Hira Farooq

**Affiliations:** ^1^Department of Pediatrics and Child Health, Aga Khan University, Karachi, Pakistan; ^2^Department of Nutrition, Aga Khan University, Karachi, Pakistan

**Keywords:** SSPE, LGIT, children, neurodegenerative, myoclonic jerks

## Abstract

**Introduction:**

Sub-acute sclerosing panencephalitis (SSPE) is a chronic, progressive neurodegenerative disorder, commonly seen in measles-endemic countries leading to progressive neuronal loss and death. Currently, there is no proven cure for this devastating disease. We started a low glycemic index therapy (LGIT) in children with SSPE using the same principle as per its role in intractable epilepsy.

**Methodology:**

Low glycemic index diet was started in children with a confirmed diagnosis of SSPE based on Dyken's criteria. All children were then classified into four stages according to disease progression. The response to diet was evaluated by improvement in their myoclonic jerks, motor activities, and changes in their stage of the disease.

**Results:**

A total of 12 children were enrolled. The mean age was 6.65 years (range 3.3–10 years), with a male-to-female ratio of 2:1. Five children were at stage IV, five were at stage III, and two were at stage II at the start of the diet. Nine (75%) children showed improvement in their stage of illness. Of three children who were at stage IV at the initiation of the diet, one improved to stage II and two to stage III. Four children at stage III reverted to stage II. Two children initiated at stage II went into total remission. Seven (58.3%) children showed a >50% reduction in myoclonic jerks with three (25%) having a 100% reduction. Three (25%) children died due to pneumonia.

**Conclusion:**

LGIT may play an effective role in the management of SSPE and gives hope to families having children with this potentially life-threatening disease.

**What this article adds:**

A novel approach for a potentially life-threatening disease.
LGIT is a more adaptable and acceptable approach in LMICs with poor educational status.

## Introduction

Sub-acute sclerosing panencephalitis (SSPE) is a chronic, progressive neurodegenerative disorder that leads to progressive neuronal loss and death (1).

The Jabbour classification characterizes SSPE by progressive cognitive decline and behavior changes followed by focal or generalized seizures as well as myoclonus, ataxia, and visual disturbance, eventually leading to the final stage of a vegetative state (1–4). SSPE is considered diagnostic if the patient fulfills at least two major and one minor Dyken's criteria (4, 5). The prognosis of SSPE is very poor, and most patients die within 1–3 years of diagnosis (4, 6). Various therapies have been attempted for treatment, but none have been proven to be effective (7).

SSPE is caused by a persistent infection or mutant measles (2). Although no prevalence data for SSPE exist, every epidemic of measles shows a rise in the cases of SSPE in Pakistan (8). Measles remains endemic in Pakistan with epidemics occurring every 2–3 years, resulting in the deaths of approximately 20,000 children annually (2, 8). While the measles conjugate vaccine (MCV1 and MCV2) coverage has improved in recent years, it is still below the recommended WHO level (9, 10). In addition to vigorous efforts for measles elimination through vaccination, it is important that we consider additional therapies for children who are already suffering from the fatal consequences of SSPE (8, 9).

A ketogenic diet (KD) has been shown to be effective in the control of intractable epilepsies (11, 12) and certain neurodegenerative disorders, i.e., Alzheimer's and Parkinson's disease (13). KD works as a neuroprotective agent by slowing or stopping neurodegeneration (13, 14). The proposed mechanisms underlying this process include the inhibition of glycolysis, resulting in the increased formation and concentration of ketone bodies, increased ATP (adenosine triphosphate) production providing more energy for the brain, decrease in free radicals, antioxidant actions (12, 13), and halting apoptosis, and stabilizing nerve-cell synapses (11, 15).

KD is a high-fat, low-carbohydrate, and moderate-protein diet which emulates the effect of fasting in producing ketosis. The classic ketogenic diet (Classic KD) comprises 90% fat and 10% carbohydrate and protein. Variation in KD distributions include modified Atkins diet (MAD), low glycemic index therapy (LGIT), and medium chain triglyceride (MCT) (12).

LGIT comprises approximately 60% fat, 30% protein, and 10% carbohydrate. The greater allowance for carbohydrates is due to the fact that the CHO foods chosen must have a glycemic index lower than 55. As foods with low glycemic index cause a lower and slower rise in blood glucose and insulin levels and lower chances of hypoglycemia, LGIT requires less strict monitoring (12, 14).

Considering the use of KD therapy as a new dimension in the management of SSPE (7, 16), we decided to start LGIT in a group of patients diagnosed with SSPE. Our main aim was to help decrease seizure frequency in these patients and assess improvement in their overall functions.

## Methodology

Children coming to the Aga Khan University and National Institute of Child Health with complaints of progressive neurodegenerative regression and myoclonic seizures were evaluated for SSPE. EEGs were conducted for each patient and were considered diagnostic if they showed classic periodic, quasiperiodic, and high-voltage slow wave complexes (Radermecker complexes) with slow background activity (17). CSF was evaluated by liquor assay using an antibody index specific for measles IgG in CSF. Values of 1.5 U/ml were considered diagnostic. Those fulfilling the above criteria were then counseled on the role of LGIT in intractable seizures.

Children were then classified into one of four stages of the disease based on the Jabbour criteria: (4, 13) “Stage I, characterized by behavioral change and cognitive decline; Stage II, which is the start of the myoclonus, gradually becoming more frequent and severe; Stage III, where various combinations of pyramidal and extrapyramidal features develop, such as rigidity, dystonia, tremor, spasticity, and hemiparesis; and Stage IV, characterized by an akinetic-mute state with episodes of drenching sweat, blood pressure fluctuation, and respiratory rate abnormalities” (4, 18).

The response to diet was evaluated with the improvement in patients' myoclonic jerks per day, improvement in motor activity, and overall responsiveness leading to a change in their stage of the disease.

After thorough counseling of parents/caregivers and a formal consent procedure, the children were started on a carbohydrate washout (CHOW) diet to help the team and family understand parental compliance with the complexity of changes required. After 2 weeks on the CHOW, the children were started on the LGIT.

Diet was calculated based on the patient's ideal body weight. The type of food was chosen after taking a dietary history from the parents on food items available to them. Calculations and recipes were then formulated accordingly. A detailed written dietary prescription was given to the families in their language of understanding. Parents with no formal education and those with a mother tongue other than Urdu had their diet explained verbally, with a translator who was preferably a close relative and understood Urdu and lived with the family. All medications were converted into tablet form. All children were started on micronutrient supplements. Families were told to strictly follow the given guidelines on sugar-free daily use substances for personal hygiene such as shampoo, soap, and toothpaste. Parents were required to maintain a record sheet (Annex A in Supplementary material) about seizure frequency, feed volume and frequency, dietary intolerance, blood sugars, and urine ketones and side effects. Parents were also requested to make home videos of the child where possible.

Investigations were conducted as per pre-established protocol; however, children from extremely poor families only underwent basic testing of a complete blood count, ALT, serum electrolytes, and uric acid. The first EEG was carried out as part of the initial diagnosis.

Follow-ups were conducted monthly either physically or through phone calls. EEGS were carried out only after considering the affordability of the patients. A change denoting improvement was considered if the high-voltage slow wave complexes became infrequent, or there were no ictal discharges associated with jerks, or the background returned to normal.

## Results

This study included 12 patients. Of these, 11 patients were confirmed for SSPE-based CSF/serum MV-specific IgG index. One child was diagnosed as probable SSPE based on the borderline-raised CSF antibody index but fulfilled all other criteria. The general characteristics of the patients are shown in Table 1.

**Table 1:** General characteristics of cases.

	**Minimum**	**Maximum**	**Mean**
Age	3 years 3 months	10 years	6.65
Gender	8 boys	4 girls	–
Ethnicity	Varied		
Duration of treatment	2 months	24 months	13 months
Medicines	2	5	3.5

Oligoclonal bands were positive in all children, and measles IgG in CSF and serum was measured in all cases, with very high CSF values in all except one patient and high serum values in all patients. EEG was diagnostic of SSPE in all patients upon initial diagnosis (Table 2).

**Table 2:** Summary table indicating individualized serology of patients with SSPE.

**S. no**	**Serum IgG measles U/ml**	**CSF IgG measles U/ml (antibody index)**	**Oligoclonal bands and type**	**Electroencephalogram (EEG) at baseline**
1.	>5,000	2.13	+ve Type 2	Intermittent high-voltage generalized delta bursts intermixed with bilateral fronto-central dominant sharp activity followed by 0.5–3 s of suppression period. Background diffuse delta activity.
2.	>5,000	1.5	+ve Type 2	Periodic burst with high amplitude with suppression, background showing diffuse delta theta activity.
3.	>5,000	1.5	+ve TYPE 2	Frequent quasi periodic high-voltage delta burst, followed by relatively low voltage activity with an interval of 2.5–4 s. Background showing diffuse theta and delta slowing.
4.	3646.1	1.67	+ve Type 3	Quasiperiodic bursts of high amplitude delta waves at intervals of 4–8 s with suppression of 0.5–2 s. Background showing diffuse delta theta activity.
5.	>5,000	1.5	+ve Type 2	Periodic bursts of high amplitude slow waves intermixed with spike and sharp waves are seen at intervals of 3–5 s, followed by 0.5–2 s of suppression period. Background showing diffuse delta theta slowing 4529476.
6.	>5,000	2.06	+ve Type 2	Frequent quasiperiodic generalized high-voltage delta slow waves interval of 3–12 s with myoclonic jerks Background showing diffuse delta theta slowing.
7.	>5,000	1.59	+ve Type 2	Periodic generalized high-voltage delta waves, burst with suppression of 0.5–1 s. Background showing diffuse theta and delta slowing.
8.	>5,000	1.5	+ve Type 2	Intermittent generalized high-voltage sharp and slow waves followed by a 2.0–4.0 s of suppression period. Background showing diffuse theta and delta slowing.
9.	>5,000	1.3	+ve Type 2	Intermittent to frequent bilateral fronto-central quasiperiodic independent (right predominant) at the time seems to be generalized high-voltage spike, sharps, and slow waves, followed by 0.5–3 s of electro decremental response without any clinical correlation. Background showing delta theta slowing.
10.	>5,000	1.68	+ve Type 3	Periodic generalized high-voltage sharp and slow waves followed by 2.0–4.0 s of suppression period. Diffuse theta and delta slowing.
11.	>5,000	1.5	+ve Type 2	A quasiperiodic build-up of rhythmic activity over the right hemisphere, followed by a sudden generalized suppression for approximately 1–1.5 s than there is a high-voltage generalized delta wave, which gradually wanes off in the next few seconds. Intermittently, right hemispheric epileptiform discharges are seen. Diffuse theta and delta slowing.
12.	>5,000	1.48 (Annex B in Supplementary material)	+ve Type 2 (Annex C in Supplementary material)	Intermittent delta theta burst intermixed with spike and wave discharges with suppression of 0.5 s. Background diffuse delta theta slowing (Supplementary Figure 2).

Parents of all children agreed to start the diet after formal consent. Two children continued CHOW after they showed improvement and parents refused to change to LGIT.

Patients' ages ranged from 3 years 3 months to 10 years at the time of dietary therapy initiation. Patients belonged to various ethnic backgrounds. This included three families that were Sindhi-speaking, three were Balochi-speaking, two were Pashto-speaking, two were Urdu-speaking, one was Punjabi-speaking, and one was Balti-speaking.

All children were malnourished at the start of the diet. In total, 75% of children were in the < 5th centile for their weight for age, while the remaining 25% were in the < 25th centile. The history of measles was observed in all children, with >90% having measles at or before 2 years of age and five patients developing it younger than 1 year of age. Six children were at stages III and IV and had a rapidly progressive disease, which had reached their current stage between 1 and 6 months.

One child was previously diagnosed with HIV and was under treatment for HIV before developing SSPE. MRIs were conducted for five children before initiating the diet. The findings were normal in three cases; one case showed multifocal hyperintense signals in T2 and flare in the cortical and subcortical areas, and the other case showed a large left temporal arachnoid cyst.

The total duration of LGIT ranged from 2 months to a maximum of 2 years with a mean of 13 months. Children, who were started on LGIT, were on a minimum of two and a maximum of five anti-seizure medications (ASM). Seven children received Isoprinosine prior to starting LGIT with no change noted (Table 1).

Five children were at stage IV, five were at stage III, and two were at stage II at the start of the diet. Nine (75%) children improved in their stages. Two of these children were at stage IV on the initiation of the diet; one improved to stage II and two improved to stage III. Four of the improved children were at stage III at the start of the diet and reverted to stage II. Two children, initially only on CHOW at stage II, went into complete remission, with no seizures or jerks and total physical independence. Two children at stage IV and one at stage III at the start of the diet showed no change in their state (Table 3).

**Table 3:** Change in stages of SSPE.

	**Stage (baseline)**	**SSPE stage after 3 months**	**SSPE stage after 6 months**
Patient 01	4	3	Died
Patient 02	4	3	3
Patient 03	4	2	3
Patient 04	3	2	Lost to follow-up
Patient 05	3	3	Lost to follow-up
Patient 06	3	2	3
Patient 07	4	4	4
Patient 08	4	4	4
Patient 09	3	2	4
Patient 10	2	0	0
Patient 11	3	3	2
Patient 12	2	0	0

Continuous myoclonic jerks and significant head drops were initially observed in all children. Seven (58%) children showed a >50% reduction in myoclonic jerks. Three (42.8%) of these seven children were in complete remission of myoclonic jerks, two of whom had completely remitted on the disease and one improved from stage IV to stage III.

Based on the number of jerks noted in motor activity and change in stage, the average duration of response to the diet was 1 month with a range of 15 days to 3 months. Blood sugar ranged between 50 and 100 mg/dl in all cases. Urinary ketones were recorded minimally with an average of +1 to +2 ketones noted.

Five children, two without and three with support, started ambulating. Two relapsed again, one after breaking the diet and the other after developing fatal pneumonia.

**Figure 1:**
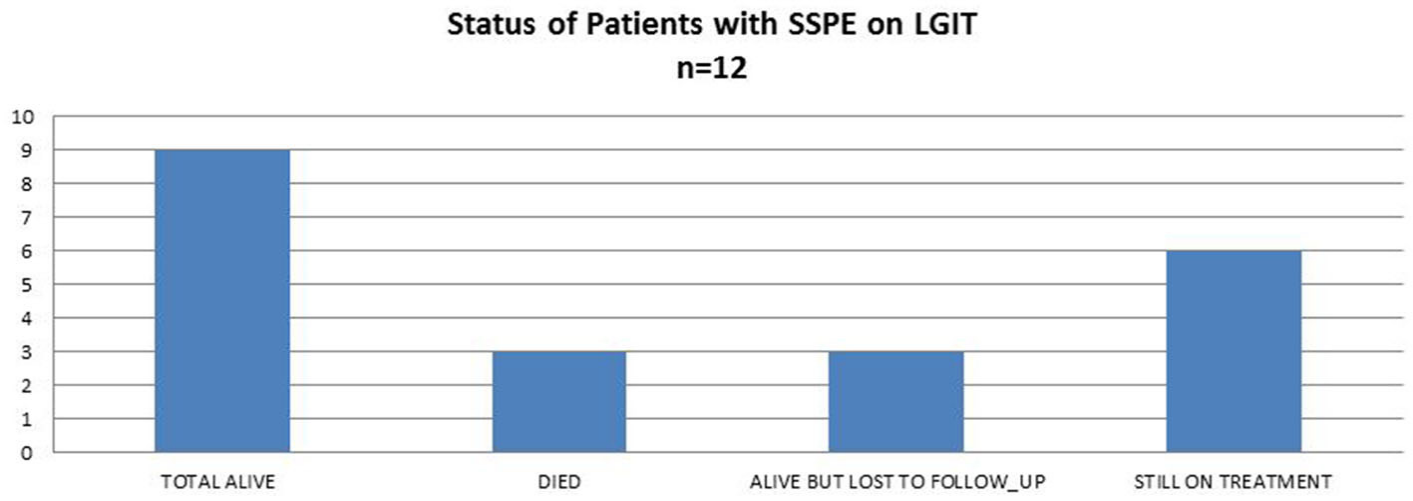
Current status of patients with SSPE on LGIT.

Three children died due to pneumonia. One of them became ambulant as mentioned above, but the other two died very early after starting treatment with no change seen during the early stages. Nine children are alive, six of whom are still on treatment, whereas three are lost to follow-up; these families were called to inquire about their status, and the children are still alive on the last call (Figure 1).

Case #2 (Table 3) is the longest survivor on the diet, whereas the shortest duration of the diet was 2 months (lost to follow-up).

Six children required admission, three due to pneumonia and two due to dehydration, requiring intravenous hydration. One child was admitted for monitoring dietary compliance and reinforcement.

A total of eight children were on an oral diet, including three children who had their NG tubes removed to start the diet. The most common complications encountered during the therapy were chest congestion and constipation. The four children on NG feeding showed better compliance as compared to those on the oral diet. In one child, the mother broke the diet by giving carbohydrates and the child regressed.

Follow-up EEGs were conducted in six children between 3 and 6 months after initiating the diet. No change was seen in five of the six children. One child went into remission and the EEG normalized (Supplementary Figure 1).

A summary of the individualized results of LGIT in children suffering from SSPE is shown in Table 4.

**Table 4:** Summary table showing individualized results of LGIT in children suffering from SSPE.

**Pt. code**	**Current status of child (dead/alive)**	**Age (at the time of LGIT initiation)**	**Gender**	**Duration of the diet**	**Status (baseline)**	**3 months follow-up**	**6 months follow-up**	**Complications /issues encountered**	**Mode of feeding**
1.	Dead	5.8 years	M	5 months	**Stage:** Stage IV **Jerks:** Whole body stiffness and continuous myoclonic jerks. **Motor activity**: Bed bound. **Speech and recognition:** No response to any vocal or physical stimulus, Bed bound. **EEG:** Intermittent high-voltage generalized delta bursts intermixed with bilateral fronto-central dominant sharp activity followed by 0.5–3 s of suppression period. Background diffuse delta activity.	**Stage:** Stage III **Jerks**: Myoclonic jerks improved by 20%. **Motor activity:** Bed bound. **Speech and recognition**: Eye-opening and movement improved on command, recognizes smiles but still bed bound. **EEG:** Semi periodic rhythmic monomorphic build-up of delta activity in a bilateral posterior quadrant, lasting for 2-3 s, followed by a brief low voltage of < 0.2 s.	Died at 5 months	Oral ulcers, constipation, pneumonia, and vomiting.	NG
2.	Alive	7 years	F	2 years	**Stage:** Stage IV **Jerks:** Continuous myoclonic jerks, stiffness, head drops. **Motor activity:** Bed bound since 1 year. **Speech and recognition:** No speech, no response. **EEG**: Periodic burst with high amplitude with suppression, background showing diffuse delta theta activity.	**Stage:** Stage III **Jerks:** Myoclonic jerks stopped within 1 month of initiating the diet. **Motor activity:** Full support sitting on a customized chair. **Speech and recognition**: No speech, responds to pain and name calling by opening eyes and turning toward the examiner. **EEG:** Not done.	**Stage:** Stage III **Jerks:** No jerks. **Motor activity:** Full support sitting on a customized chair. **Speech and recognition:** No speech, respond to pain and name calling. **EEG** Periodic burst suppression intermittent spike and waves no ictal record noted.	Bedsore grade IV Pneumonia and repeated Chest congestion, vomiting, dehydration. Admitted to the hospital for rehydration.	NG feeding
3.	Alive (on Treatment for HIV)	4 years	M	1 year 11 months	**Stage:** Stage IV **Jerks:** Continuous head drops, full body jerks (100/day), eye up rolling, no neck holding. **Motor activity:** Bed bound. **Speech and recognition**: No speech. **EEG:** Frequent quasi periodic high-voltage delta burst, followed by relatively low voltage activity with an interval of 2.5–4 s. Background showing diffuse theta and delta slowing.	**Stage:** Stage II **Jerks:** 3–4 head drops/day. Whole body jerks decreased by 50%. **Motor activity:** Start support sitting, standing with support. **Speech and recognition:** Started talking and responding to questions could give his place of residence when asked nasogastric tube removed with full oral intake. EEG not done.	**Stage:** Stage III (relapsed) **Jerks:** Head drops 1–2/min **Motor activity:** Support sitting, standing. **Speech and recognition:** Stopped talking again after breaking the diet. On restarting, jerks have stopped and can only sit with support and no speech. **EEG:** Slow background. Intermittent to infrequent burst of high amplitude burst followed by a slow period of 0.5 s intermixed with spikes. No ictal discharges/.	Relapse due to non-compliance pneumonia and recurrent chest congestion, fever, admitted for non-compliance management. Non-compliance with the diet by the mother (mother gave roti after 2 months of diet, mother language barrier, lack of mother's understanding)	Initially on NG feeding, After 2 months Ng removed now on oral diet.
4.	Alive	5 years	M	5 Months of therapy then lost to follow-up	**Stage:** Stage III **Jerks:** Uncontrolled whole body jerks continuous head drops, abnormal eye movement. **Motor activity:** No neck holding, bed bound. **Speech and recognition:** No speech, no response. **EEG:** Quasiperiodic bursts of high amplitude delta waves at intervals of 4–8 s with suppression of 0.5–2 s. Background showing diffuse delta theta activity.	**Stage:** Stage II **Jerks:** Whole body jerks improved by 25%. After 3 months, no jerks during sleep, only brief in the awake state. **Motor activity:** Support sitting, head control improved. **Speech and recognition**:, More alert, smiles, focusing. No EEG done.	Lost to follow-up after 5 months of diet. No EEG done.	Lost to Follow-up. Chest congestion, vomiting. Language barrier.	Oral
5.	Alive	3.3 years	M	2 Months of therapy then lost to follow-up	**Stage:** Stage III **Jerks:** Continuous head drops, continuous whole body jerks while awake. **Motor activity:** No neck holding, bed bound. **Speech and recognition:** No speech, no response. **EEG:** Periodic bursts of high amplitude slow waves intermixed with spike and sharp waves are seen at intervals of 3–5 s, followed by 0.5–2 s of suppression period. Background showing diffuse delta theta slowing 4529476.	**Stage:** Stage III**Jerks:** 10 % decrease in myoclonic jerks **Motor activity:** Neck holding improved, support sitting. **Speech and recognition**: No speech, response to pain only. **EEG:** Not done.	Lost to follow-up after 2 months of therapy.	Vomiting, loose stools. Lost to follow-up after 2 months. Language barrier, Lack of mother's understanding, family not supportive.	On Ng feed then Ng removed.
6.	Dead	8 years	M	1 year 2 months	**Stage:** Stage III **Jerks**: Whole body stiffness, continuous myoclonic jerks, > 150/day **Motor activity:** Support sitting and standing. **Speech and recognition:** Delayed verbal response. **EEG:** Frequent quasiperiodic generalized high-voltage delta slow waves interval of 3–12 s with myoclonic jerks. Background showing diffuse delta theta slowing.	**Stage:** Stage II Then, regressed after pneumonia in 1 month. **Jerks:** Myoclonic jerks decreased by 40 % in 1 month. Relapsed again after a month. **Motor activity**: Bed bound, support sitting. **Speech and recognition:** Started talking after 1 week of diet but after relapse speech lost, no response, increased crying with a loud voice.**EEG:** Not done.	**Stage**: Stage III (died after 1 year) **Jerks:** Uncontrolled continuous whole body jerks. **Motor activity:** Bed bound **Speech and recognition:** No speech, respond to pain only. **EEG:** Not done.	Admitted in Peshawar with pneumonia, long distance management repeated chest congestion, constipation.	On oral feed then NG placed.
7.	Alive	5 years	M	7 months therapy, lost to follow-up	**Stage:** Stage IV **Jerks**: Myoclonic jerks every 2–3 sec. No neck holding, uncountable head drops. **Motor activity:** Bed bound. **Speech and recognition:** No verbal response. **EEG:** Periodic generalized high-voltage delta waves, burst with suppression of 0.5–1 s, background showing diffuse theta and delta slowing.	**Stage:** Stage IV **Jerks:** After 2 months of diet, 50% improvement in jerks and head drops. **Motor activity:** No neck holding, bed bound. **Speech and recognition:** No speech, no response. **EEG:** Not done.	**Stage:** Stage IV (lost to follow-up after 7 months). **Jerks:** Remains at 50% improvement in jerks. **Motor activity:** Bed bound. **Speech and recognition:** No speech, no response. **EEG:** Not done.	Lost to follow-up after 7 months pneumonia, low socioeconomic level, poor literacy level, loss of interest in the diet, parents became exhausted. Constipation, chest congestion.	NG
8.	Alive	10 years	F	11 months	**Stage:** Stage IV **Jerks:** Whole body stiffness, tremors, continuous myoclonic jerks, uncontrolled arm movements. **Motor activity**: Bed bound. **Speech and recognition:** Not responsive. Severely malnourished **EEG:** Intermittent generalized high-voltage sharp and slow waves followed by a 2.0–4.0 s of suppression period. Background showing diffuse theta and delta slowing.	**Stage:** Stage IV **Jerks:** After 1 month of diet, body stiffness improved, tremors decreased but were not recorded, decreased myoclonic jerks by 20%. **Motor activity:** Bed bound. **Speech and recognition:** Eye opening on pain. **EEG:** Not done.	**Stage:** Stage IV **Jerks:** Remains at 20% improvement in myoclonic jerks. **Motor activity:** Bed bound **Speech and recognition:** No speech, respond to pain only. **EEG:** Not done.	Weight loss and dehydration admitted for rehydration and dietary monitoring, constipation, pneumonia, weight loss low socioeconomic level, lost to follow-up after 5 months because of cost and reverted after being called.	NG
9.	Dead	4.8 years	M	7 months	**Stage:** Stage III **Jerks**: Head drops, whole body myoclonic jerks 100 per day. **Motor activity:** Support sitting. **Speech and recognition:** No speech **EEG:** Intermittent to frequent bilateral fronto-central quasiperiodic independent (right predominant) at time seems to be generalized high-voltage spike, sharp and slow waves, followed by 0.5–3 s of electro decremented response without any clinical correlation. Background showing delta theta slowing.	**Stage:** Stage II **Jerks**: Decreased jerks by 50%. **Motor activity:** Started support walking after 1.5 months of diet. **Speech and recognition:** No speech but responds to pain and name calling. **EEG:** Not done.	**Stage:** Stage IV (relapsed) Diet at 7 months. **Jerks:** Remains at 50 % improvement in jerks. **Motor activity:** Bed bound. **Speech and recognition:** No speech. **EEG:** Not done.	Pneumonia, dehydration, chest congestion, and vomiting.	Oral
10.	Alive	9 years	F	1 year 4 months	**Stage:** Stage II **Jerks:** Uncontrolled head drops, jerks, mild eye twitching. **Motor activity:** Support standing and walking. **Speech and recognition:** Talking but with a delayed response. **EEG:** Periodic generalized high-voltage sharp and slow waves followed by a 2.0–4.0 s of suppression period. Diffuse theta and delta slowing.	**Stage:** Stage 0 **Jerks**: After 15 days of CHOW 50% decrease in head drops, after a month, no head drops, myoclonic jerks stopped 100%. fMotor activity: Walking without support. **Speech and recognition:** Talking and more responsive. **EEG:** Not done.	**Stage:** Stage 0 **Jerks:** No jerks. **Motor activity:** Walking without support, looking after own bodily needs but with assistance. **Speech and recognition:** Full speech and communication. **EEG:** Not done.	Issues with oral intake, food choices, difficulty to start LGIT.	Oral (CHOW)
11.	Alive	5 years	M	1 year 5 months	**Stage:** Stage III **Jerks:** Continuous jerks, whole body stiffness. **Motor activity:** No neck holding, bed bound. **Speech and recognition:** No speech. **EEG:** Quasiperiodic build-up of rhythmic activity over the right hemisphere, followed by a sudden generalized suppression for approximately 1–1.5 s than there is a high-voltage generalized delta waves, which gradually wane off in next few seconds. Intermittently, right hemispheric epileptiform discharges seen. Diffuse theta and delta slowing.	**Stage**: Stage III **Jerks:** Myoclonic jerks decreased by 60%. **Motor activity:** Support sitting. **Speech and recognition:** Response improved. After 1 month of diet and after 4 months of diet:	**Stage:** Stage II **Jerks:** Remains at 60 % decrease. **Motor activity:** Support standing and neck holding improved. **Speech and recognition:** Following commands. **EEG:** Continuous quasiperiodic high-voltage generalized delta bursts intermixed with spike, polyspike, sharp and slow waves, followed by 1.5–3.5 s of suppression period, clinically associated with myoclonic jerks.	Constipation, pneumonia, vomiting, fatty stools.	Initially oral, now on NG feed after pneumonia.
12.	Alive	10 years	F	4 Months	**Stage:** Stage II **Jerks:** Whole body jerks, uncontrolled head drops. **Motor activity:** Walking with support but frequent episodes of fall. **Speech and recognition:** Delayed verbal response. **EEG:** (Supplementary Figure 2). Intermittent delta theta burst intermixed with spike and wave discharges with suppression of 0.5 s. Background diffuse delta theta slowing.	**Stage**: Stage 0 **Jerks:** Decreased head drops 10–12 per h after 15 days of CHOW. Myoclonic jerks stopped by 100 % after 2 months. **Motor activity:** No episode of fall, started walking without support. **Speech and recognition:** Full conversation.	**Stage:** Stage 0 **Jerks:** No jerks. **Motor activity:** Waking without support, looking after own bodily needs but with assistance. **Speech and recognition:** Full conversation. **EEG:** (Supplementary Figure 1). Normal awake EEG showing a background of 8–9 Hz and no burst of delta theta waves. No spike and wave discharges seen.	Issues with oral intake, food choices, difficulty to start LGIT low socioeconomic level.	Oral (CHOW)

## Discussion

Early onset of measles is strongly associated with the development of SSPE (15), the most common neurodegenerative disorder in Pakistan. While, in recent years, the experience of developed countries with SSPE mortality characterizes it as a “vanishing disease,” (6) measles-endemic countries experience SSPE as the most common post-infectious neurodegenerative cause of mortality in children (19).

SSPE is entirely preventable by vaccination but is still prevalent in Pakistan due to low acceptance and coverage of the vaccine. Unpublished data from a large pediatric public sector hospital in Karachi with a specific pediatric neurology section report the average number of SSPE cases to be approximately 8–10 per month. A 1988 study from Pakistan reported that SSPE represented approximately 10% of inflammatory afflictions of the cerebral parenchyma, and its incidence rate was approximately 100 times more than that observed in developed countries (19). A 2014 study by Ibrahim et al. also showed an increase in the cases reported with every epidemic of measles (8, 9).

This study details the preliminary data of 12 children who, after the initiation of LGIT, showed a difference in both the control of myoclonic jerks and overall improvement in the stage of SSPE.

A systematic review conducted in India on treatment options for SSPE has indicated that KD or its various types hold the potential to be incorporated into future SSPE treatment plans (20). Another multicenter review also mentions KD as a possible option in the treatment of SSPE (7). Two case reports from India and USA have used classic KD in children with SSPE. One study reported almost complete remission of the disease in a child with stage IIIa on classic KD with improvement in cognitive as well as physical activity in 3 months (15). The other reported the case of a child showing initial improvement in myoclonic jerks after classic KD but regressing again after a few months (16).

However, a practical issue for LMICs is that classic KD is associated with higher non-compliance (21). Our own previous experience (unpublished) with KD in untraceable epilepsies also showed poor compliance with the classic KD. The cost of the classic KD often becomes devastating to most families (21). This is why, we chose to start the LGIT type of KD in our patients.

All children in our study had a history of measles before or at 2 years; however, this did not alter the response to treatment. Overall, eight (63.6%) children showed >50% reduction in myoclonic jerks, and nine (75%) children improved in their stages of disease. However, the ones not showing a significant change were the ones lost to follow-up, and we assume that, despite starting LGIT, they were also non-compliant. It is interesting to note that one patient who was in stage IV for 1 year before the start of the diet showed a response of almost 100% control in myoclonic jerks and improvement in stage III and is currently the longest survivor, giving hope that this diet may be effective, despite the stage and duration of the disease.

Two children in our study who were in stage II showed complete remission only with the CHOW. This suggests that the earlier the diet is started, the better the chances of complete remission. Both these families refused to change to LGIT despite improvement. In poor, uneducated families, compliance with a strict diet is difficult (21). CHOW seemed more acceptable and easier to manage for these families.

Mortality rates for SSPE are generally high, with death occurring within 1–3 years of the onset (2). The longest reported remission is an individual who was diagnosed at 17 years and, after initially regressing, stabilized and went into spontaneous remission with no clear explanation (22). In comparison, mortality was 25% in our study. Although the time frame of our study was only 2 years, 75% of children are still surviving, giving some hope in an endemic society like Pakistan.

Pneumonia and recurrent chest congestion were the most common complications seen in our patients. The three children who died also had severe pneumonia and eventually respiratory failure. One of these children was ventilated, and the LGIT was changed to an intravenous formulation with protein and fat calculated as per the LGIT. He showed improvement initially, however, after 1 month, he had another bout of pneumonia at home and eventually died. KD is associated with recurrent pneumonia and aspiration, which has been related to high lipid content or lower immunity (23, 24). However, the commonest complication with SSPE is also reported to be pneumonia, and at present, it is unclear whether this could be due to the primary condition or a complication of the diet (25). Children in stages III and IV are either on NG feeding or are being fed by parents forcibly. This may result in aspiration pneumonia, causing regression in the achieved improvement. A study from Lahore, Pakistan, states that malnutrition and poor socioeconomic status may be the predictors of a higher risk of complications for children with SSPE (26).

The results seen in this case series are promising and suggest that LGIT can play a beneficial role in the management of SSPE. LGIT is specifically a more practical option in lower- and middle-income countries (LMICs), where the affordability, adaptability, and acceptability of LGIT are comparatively higher than classic KD.

## Conclusion

Vaccination remains the only way that the potentially fatal SSPE can be eradicated. However, for pediatric neurologists, treating children with this devastating disease is extremely challenging. The findings of these cases may serve as hope for the improvement in the quality of life of patients suffering from SSPE.

A controlled trial with better follow-ups comparing both LGIT and the classic KD would be helpful to reach a conclusion as to which specific diet would be more beneficial.

## Limitations

Due to limited resources, we were unable to conduct strict follow-ups and repeat investigations as required. This is the first study of its kind, and even though there was a limited number of patients, there was a demonstrated improvement. However, a full trial with strict control would be more advantageous.

Being a potentially fatal condition, it was not possible to do a randomized controlled trial for this study.

## Data availability statement

The original contributions presented in the study are included in the article/Supplementary material, further inquiries can be directed to the corresponding author.

## Ethics statement

The studies involving human participants were reviewed and approved by 2023-8550-24416. SI: Low Glycemic Index Therapy in Sub Acute Sclerosing Panencephalitis (SSPE): An Experience from a Measles endemic country. ERC Aga Khan University. Written informed consent to participate in this study was provided by the participants' legal guardian/next of kin.

## Author contributions

SI did the conception of the study and edited the manuscript. SI and HF contributed equally to the design, collection of the literature, table preparation, and writing of the manuscript. Both authors contributed substantially to the writing and revision of the manuscript.

## Supplementary material

Supplementary Figure 1 EEG after CHOW patient #12.

Supplementary Figure 2 EEG at start of therapy patient #12.

## References

[1] Rafique A Amjad N Chand P Zaidi SSZ Rana MS Ahmed K. Subacute sclerosing panencephalitis: clinical and demographic characteristics. J Coll Phys Surg Pak. (2014) 24:557–60. 25149833

[2] Jafri SK Kumar R Ibrahim SH. Subacute sclerosing panencephalitis-current perspectives. Pediatr Health Med Ther. (2018) 9:67–71. 10.2147/PHMT.S126293 29985487 PMC6027681

[3] Jabbour J Duenas D Modlin J. SSPE-clinical staging, course, and frequency. Arch Neurol. (1975) 32:493–4.

[4] Dyken PR. Neuroprogressive disease of post-infectious origin: a review of a resurging subacute sclerosing panencephalitis (SSPE). Ment Retard Dev Disabil Res Rev. (2001) 7:217–25. 10.1002/mrdd.1030 11553938

[5] Almeida KJ Brucki SMD Duarte MIS Pasqualucci CA Rosemberg S Nitrini R. Basal ganglia lesions in subacute sclerosing panencephalitis. Dement Neuropsychol. (2012) 6:286–9. 10.1590/S1980-57642012DN06040014 29213810 PMC5619342

[6] Garg R. Subacute sclerosing panencephalitis. Postgrad Med J. (2002) 78:63–70. 10.1136/pmj.78.916.63 11807185 PMC1742261

[7] Samia P Oyieke K Tunje D Udwadia-Hegde A Feemster K Oncel I. Options in the treatment of subacute sclerosing panencephalitis: implications for low resource areas. Curr Treat Options Neurol. (2022) 24:99–110. 10.1007/s11940-022-00710-x 35340572 PMC8933242

[8] Ibrahim SH Amjad N Saleem AF Chand P Rafique A Humayun KN. The upsurge of SSPE-a reflection of national measles immunization status in Pakistan. J Trop Pediatr. (2014) 60:449–53. 10.1093/tropej/fmu050 25232151 PMC4303770

[9] Mere MO Goodson JL Chandio AK Rana MS Hasan Q Teleb N. Progress toward measles elimination-Pakistan, 2000-2018. MMWR Morb Mortal Wkly Rep. (2019) 68:505. 10.15585/mmwr.mm6822a4 31170125 PMC6553804

[10] Hasan Q Bosan A Bile K. A review of EPI progress in Pakistan towards achieving coverage targets: present situation and the way forward. East Mediterr Health J. (2010) 16(Suppl):S31–8. 10.26719/2010.16.Supp.31 21495586

[11] Rho JM. How does the ketogenic diet induce anti-seizure effects? Neurosci Lett. (2017) 637:4–10. 10.1016/j.neulet.2015.07.034 26222258

[12] D'Andrea Meira I Romão TT Pires do Prado HJ Krüger LT Pires MEP da Conceição PO. Ketogenic diet and epilepsy: what we know so far. Front Neurosci. (2019) 13:5. 10.3389/fnins.2019.00005 30760973 PMC6361831

[13] Włodarek D. Role of ketogenic diets in neurodegenerative diseases (Alzheimer's disease and Parkinson's disease). Nutrients. (2019) 11:169. 10.3390/nu11010169 30650523 PMC6356942

[14] Liśkiewicz A Jedrzejowska-Szypułka H Lewin-Kowalik J. Characteristics of ketogenic diet and its therapeutic properties in central nervous system disorders. Ann Acad Med Siles. (2012) 6:66–76.

[15] Nathan J Kale DK Naik VD Bailur S. Substantial remission in subacute sclerosing panencephalitis by following the ketogenic diet: a case report. Cureus. (2019) 11:e5485. 10.7759/cureus.5485 31489275 PMC6713239

[16] Bautista RED. The use of the ketogenic diet in a patient with subacute sclerosing panencephalitis. Seizure. (2003) 12:175–7. 10.1016/S1059-1311(02)00268-6 12651085

[17] Gutierrez J Issacson RS Koppel BS. Subacute sclerosing panencephalitis: an update. Dev Med Child Neurol. (2010) 52:901–7. 10.1111/j.1469-8749.2010.03717.x 20561004

[18] Saurabh K Singh VK Pathak A Chaurasia RN. Subacute sclerosing pan encephalitis: an update. J Clin Sci Res. (2021) 10:35–42. 10.4103/JCSR.JCSR_68_2034988262

[19] Kondo K Takasu T Ahmed A. Neurological diseases in Karachi, Pakistan-elevated occurrence of subacute sclerosing panencephalitis. Neuroepidemiology. (1988) 7:66–80. 10.1159/000110138 3374729

[20] Pritha A Medha TN Garg RK. A comprehensive investigation of the current subacute sclerosing panencephalitis (SSPE) treatment options to improve patient quality of life. Cureus. (2022) 14:e28389. 10.7759/cureus.28389 36171840 PMC9508860

[21] Ye F Li X-J Jiang W-L Sun H-B Liu J. Efficacy of and patient compliance with a ketogenic diet in adults with intractable epilepsy: a meta-analysis. J Clin Neurol. (2015) 11:26–31. 10.3988/jcn.2015.11.1.26 25628734 PMC4302176

[22] Santoshkumar B Radhakrishnan K. Substantial spontaneous long-term remission in subacute sclerosing panencephalitis (SSPE). J Neurol Sci. (1998) 154:83–8. 10.1016/S0022-510X(97)00303-1 9543327

[23] Buda P Wieteska-Klimczak A Własienko A Mazur A Ziołkowski J Jaworska J. Lipoid pneumonia-a case of refractory pneumonia in a child treated with ketogeic diet. Adv Respir Med. (2013) 81:448–52. 10.5603/ARM.35520 23996884

[24] Woody RC Steele RW Knapple WL Pilkington NS Jr. Impaired neutrophil function in children with seizures treated with the ketogenic diet. J Pediatr. (1989) 115:427–30. 10.1016/S0022-3476(89)80847-9 2769501

[25] Rinawati W Kumalawati J. Oligoclonal bands: a laboratory diagnosis of subacute sclerosing panencephalitis (SSPE). J Infect Dev Ctries. (2022) 16:1096–100. 10.3855/jidc.15200 35797306

[26] Malik MA Saeed M Qureshi AU Ahmed N Akram M. Predictors of clinical course of subacute sclerosing panencephalitis: experience at the Children's Hospital, Lahore. J Coll Phys Surg Pak. (2010) 20:671–4. 20943110 10.2010/JCPSP.671674

