# Supplementary material for: Low glycemic index therapy in children with sub-acute sclerosing panencephalitis (SSPE): an experience from a measles-endemic country
Source: Front Nutr. 2023 Jul 24;10:1203144. doi: 10.3389/fnut.2023.1203144 (PMC10406380; doi:10.3389/fnut.2023.1203144)
Supplement: Supplementary file 1 [file Data_Sheet_1.zip › ANNEX A.DOCX]

ANNEX A

**Record Sheet**

| **Date** | **No of Seizures/ Jerks** | **Duration** | **Blood Sugars** | **Urine ketones** | **Bowel Movements** | **/ Food Feed**  **Frequency** | **Vomit** | **Fluid** | **Sleep** |
| --- | --- | --- | --- | --- | --- | --- | --- | --- | --- |
|  |  |  |  |  |  |  |  |  |  |
