# Supplementary material for: Low glycemic index therapy in children with sub-acute sclerosing panencephalitis (SSPE): an experience from a measles-endemic country
Source: Front Nutr. 2023 Jul 24;10:1203144. doi: 10.3389/fnut.2023.1203144 (PMC10406380; doi:10.3389/fnut.2023.1203144)
Supplement: Supplementary file 1 [file Data_Sheet_1.zip › ANNEX B.DOCX]

ANNEX B

**Serum IgG Measles**

Cut-off Value:

<200 U/ ml: Negative

200-250 U/ ml: Intermediate

>250 U/ ml 1: Positive

**CSF IgG Measles:**

AI <1.3 U/ ml: Normal Range

AI 1.3-1.5 U/ ml: Intermediate

AI > 1.5 U/ ml: Intrathecal synthesis of specific measles virus antibody
