# Supplementary material for: Low glycemic index therapy in children with sub-acute sclerosing panencephalitis (SSPE): an experience from a measles-endemic country
Source: Front Nutr. 2023 Jul 24;10:1203144. doi: 10.3389/fnut.2023.1203144 (PMC10406380; doi:10.3389/fnut.2023.1203144)
Supplement: Supplementary file 1 [file Data_Sheet_1.zip › ANNEX C.DOCX]

ANNEX C: Types of Oligoclonal Bands (OCBs)

**“Type 1 pattern** = absence of OCBs in serum and CSF

**Type 2 Pattern** = presence of OCBs in CSF

**Type 3 Pattern** = presence of OCBs in CSF and additional identical OCBs in both serum and CSF

**Type 4 pattern** = presence of identical OCBs in both serum and CSF and results indicating Intrathecal IgG synthesis “
