# Supplementary figures and images for: Low glycemic index therapy in children with sub-acute sclerosing panencephalitis (SSPE): an experience from a measles-endemic country
Source: Front Nutr. 2023 Jul 24;10:1203144. doi: 10.3389/fnut.2023.1203144 (PMC10406380; doi:10.3389/fnut.2023.1203144)

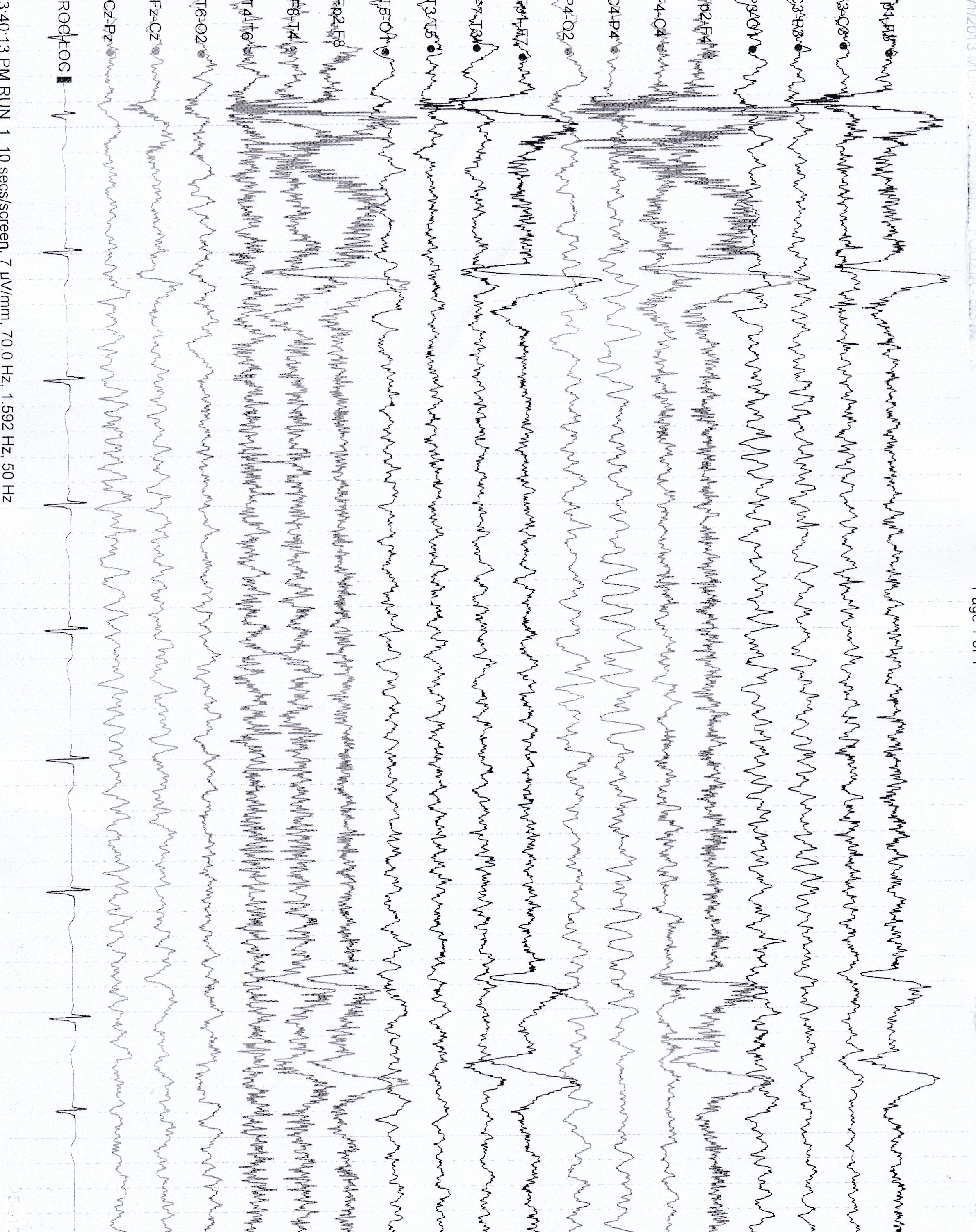

Supplement: Supplementary Figure 1 — EEG after CHOW patient #12. [file Image_1.jpg]

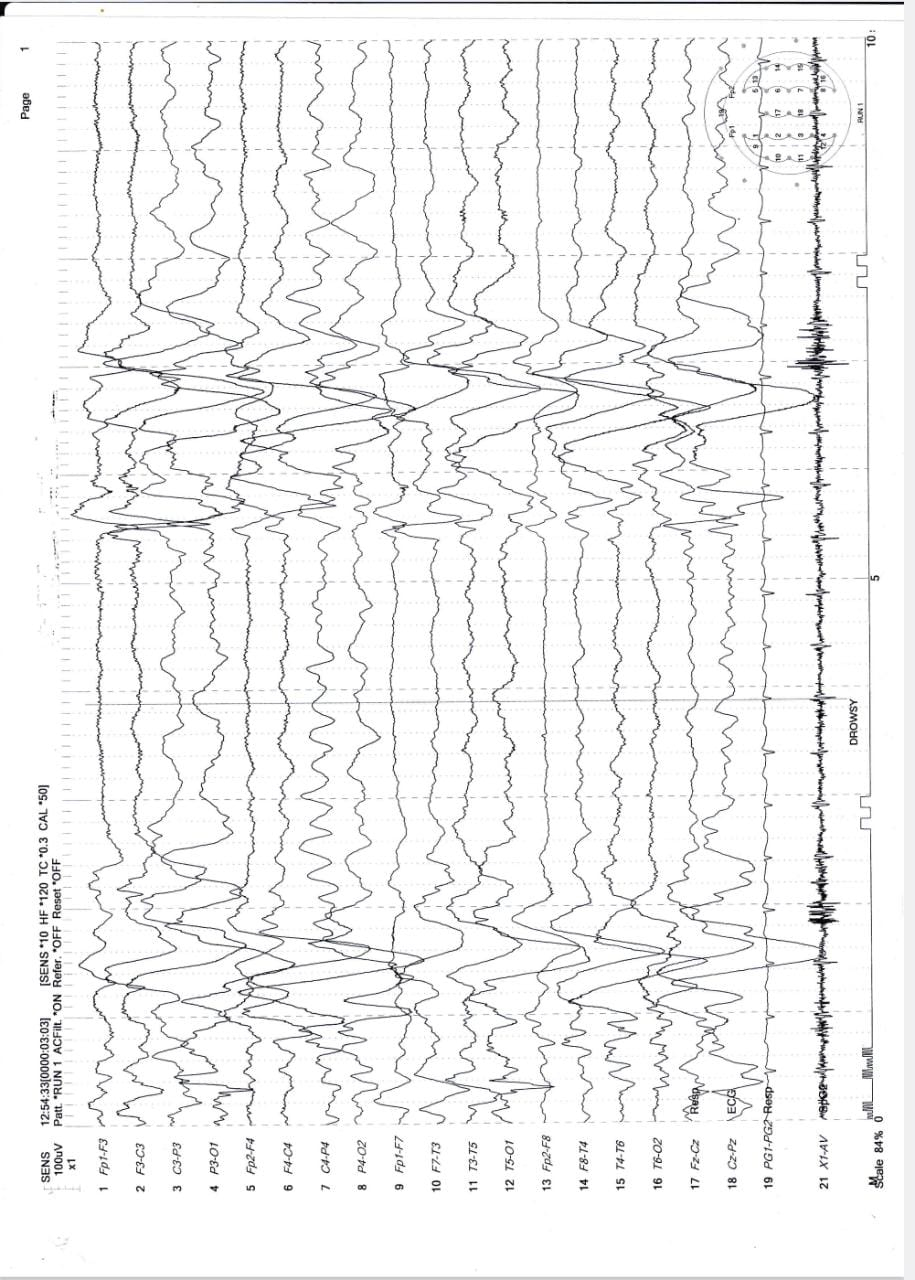

Supplement: Supplementary Figure 2 — EEG at start of therapy patient #12. [file Image_2.jpg]
